# Urothelial Carcinoma of the Bladder Following BK Virus Infection in a Pediatric Kidney Transplant Recipient

**DOI:** 10.1111/petr.70303

**Published:** 2026-03-19

**Authors:** Martina Ichas, Lise Allard, Luke Harper, Mokrane Yacoub, Iona Madden, Cécile Vérité, Astrid Godron‐Dubrasquet, Jérôme Harambat

**Affiliations:** ^1^ Service de Néphrologie Pédiatrique, CHU de Bordeaux Bordeaux France; ^2^ Service de Chirurgie Pédiatrique, CHU de Bordeaux Bordeaux France; ^3^ Service de Pathologie, CHU de Bordeaux Bordeaux France; ^4^ Service D'hémato‐Oncologie Pédiatrique, CHU de Bordeaux Bordeaux France

**Keywords:** BK virus, bladder cancer, pediatric kidney transplantation, urothelial carcinoma

## Abstract

**Background:**

Urothelial bladder carcinoma is extremely rare in children and its association with BK virus infection remains unclear.

**Methods:**

We describe the case of an 11‐year‐old girl who developed a urothelial carcinoma of the bladder four years after receiving her first kidney transplant. Kidney failure was secondary to nephronophthisis (*NPHP6* variant), diagnosed in the neonatal period and associated with Leber congenital amaurosis and intellectual disability. She underwent peritoneal dialysis for four years before kidney transplantation at 6.5 years of age. Five months post‐transplant, she developed BK virus‐associated nephropathy, leading to chronic allograft dysfunction. Four years later, a routine ultrasound revealed an asymptomatic bladder mass without evidence of extension. The lesion was resected endoscopically and later managed with partial cystectomy.

**Results:**

Histopathologic analysis confirmed a high‐grade invasive urothelial carcinoma (pT2). Immunohistochemistry showed SV40 positivity, consistent with BK virus‐induced neoplasia, while non‐tumoral cells were negative. BK viremia had been undetectable one year prior to diagnosis. The patient remained disease‐free for seven years following surgery, without adjuvant therapy.

**Conclusion:**

The involvement of BK virus in the development of bladder cancer has not yet been clarified. This case supports a possible role of BK virus in urothelial tumorigenesis, particularly in immunosuppressed transplant recipients.

## 1 Case report

Urothelial bladder carcinoma is exceptionally rare in the pediatric population, representing less than 0.01% of cancer cases under 20 years old, with known risk factors including tobacco and exposure to carcinogens (aromatic amine) in adults [1, 2, 3]. Although the BK virus has been investigated as a potential oncogenic agent, particularly in immunosuppressed patients, its causal role remains unclear.

We report the case of an 11‐year‐old girl with nephronophthisis secondary to an *NPHP6* pathogenic variant, which was associated with chronic kidney disease, intellectual disability, and Leber congenital amaurosis. Her kidney function deteriorated rapidly from the neonatal period, progressing to end‐stage kidney disease requiring peritoneal dialysis by 2 years of age. She received a deceased donor kidney transplant at the age of 6.5. Initial immunosuppression included tacrolimus, mycophenolate mofetil, and prednisolone.

Five months following transplantation, she developed a biopsy‐confirmed BK virus‐associated nephropathy resulting in chronic allograft nephropathy. BK virus nephropathy was managed by reducing tacrolimus, withdrawal of mycophenolate mofetil while continuing prednisolone, and initiating antiviral therapy with leflunomide.

**FIGURE 1:**
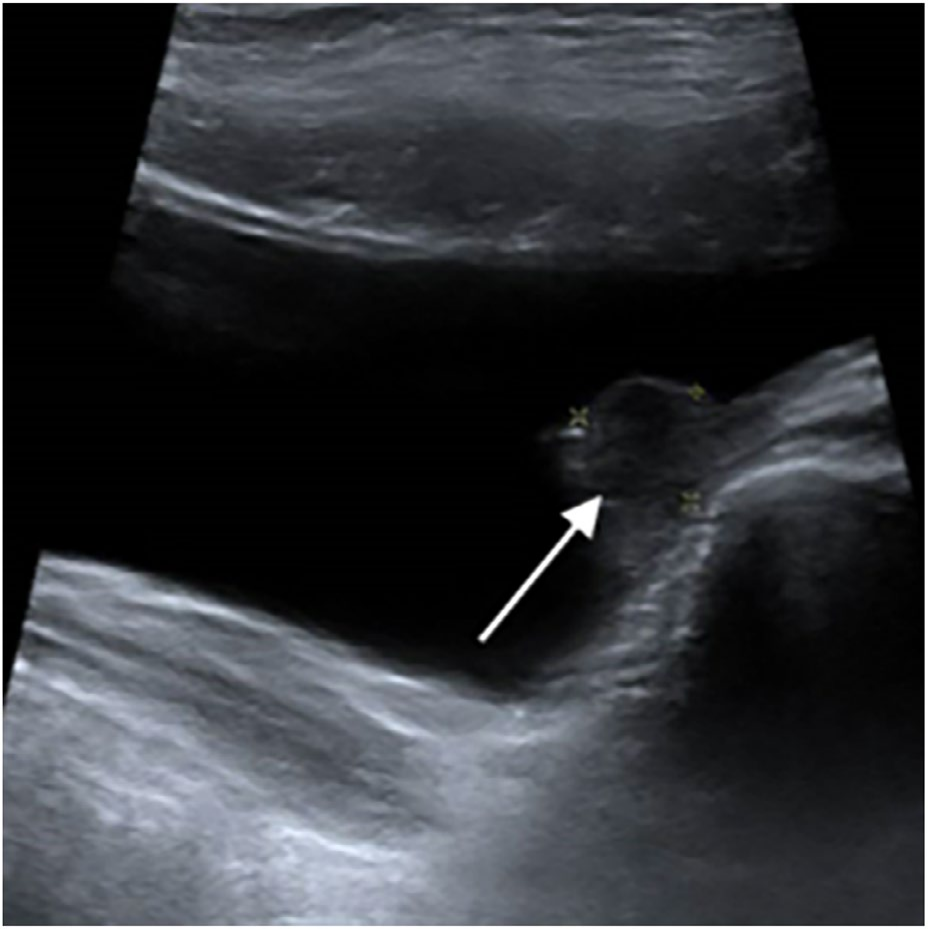
Ultrasonography with a 10 mm nodule at the left posterolateral wall of the bladder (white arrow).

**FIGURE 2:**
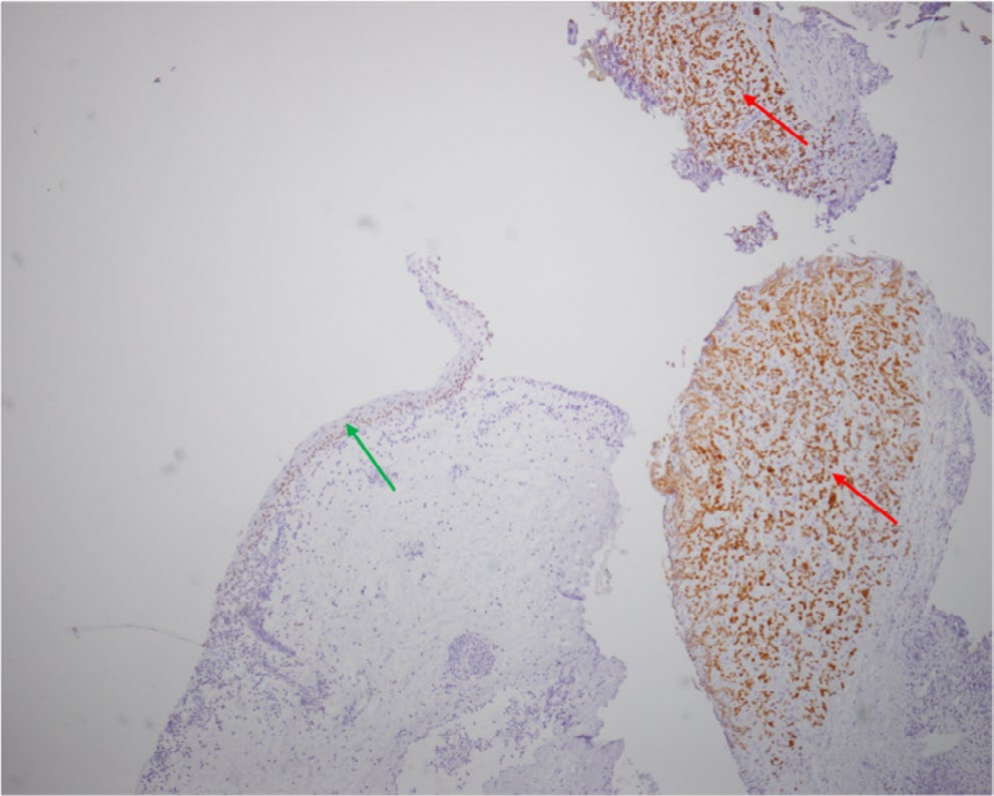
Immunohistochemical reaction with GATA3 (original magnification × 40), showing strong positivity of tumoral urothelial cells (red arrow) and positivity of the normal urothelial epithelial layer (green arrow).

Four years after transplantation, whereas the patient was asymptomatic with decreased graft function (GFR 20 mL/min/1.73 m^2^) and BK viral DNA PCR negative for at least one year *(BKV status in urine unknown)*, a routine ultrasound screening showed a 10 mm nodule in the left posterolateral wall of the bladder (Figure 1). Initially, a simple biopsy revealed a transitional cell carcinoma proliferation positive for CK7 and GATA3 (Figure 2), suggestive of a urothelial carcinoma. BKV DNA in blood was still negative at the time of diagnosis. Transurethral resection was then performed, confirming high‐grade poorly differentiated urothelial carcinoma (stage pT2). No tumor extension was found (thoracic CT‐scan, abdominal MRI, PET‐scan).

**FIGURE 3:**
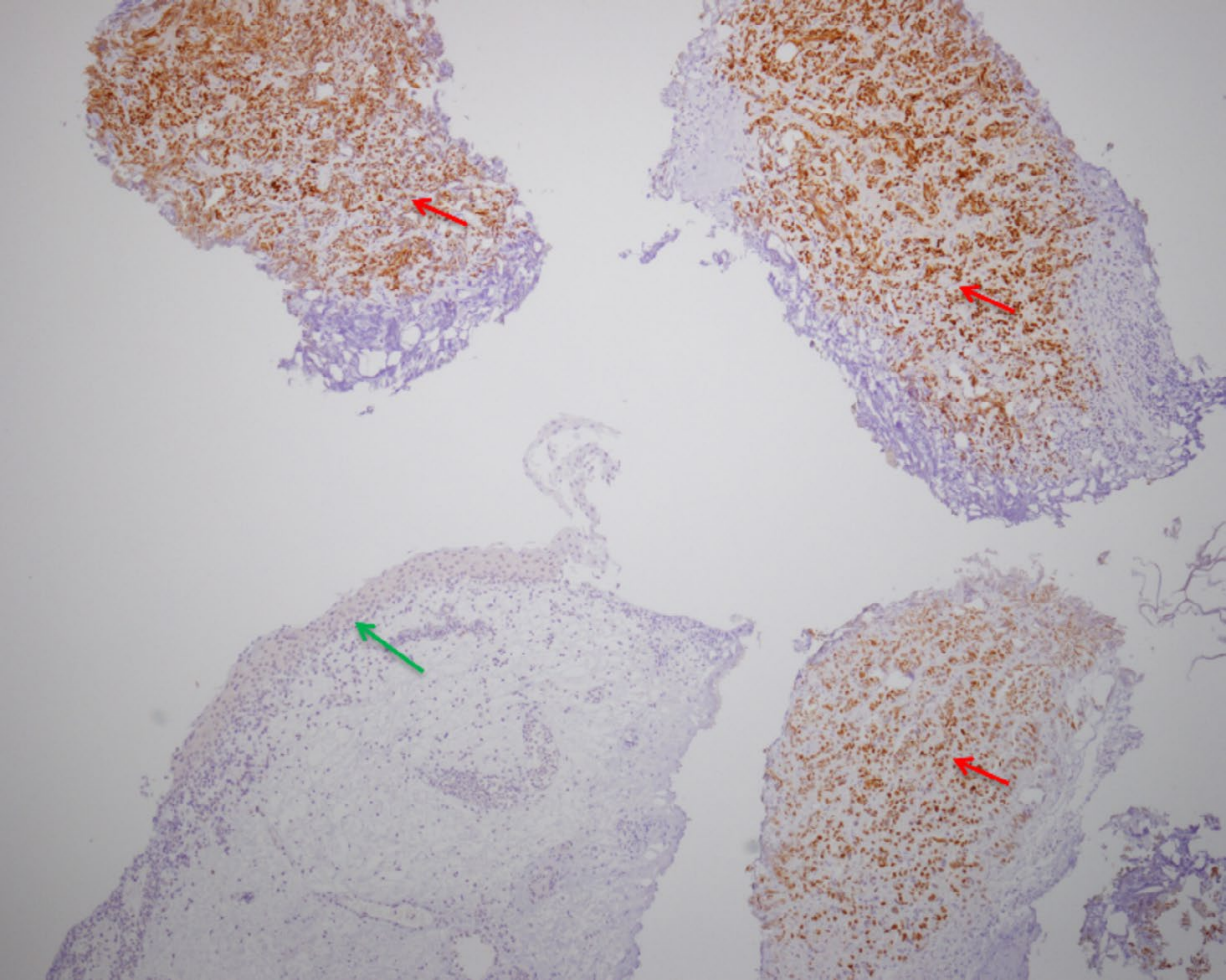
Immunohistochemical reaction with SV 40 (original magnification × 40), showing strong positivity of tumoral cells (red arrow) and negativity of the normal urothelial layer (green arrow).

The patient then underwent partial wedge‐resection cystectomy, sparing the ureters. Histological examination revealed a selective immunohistochemical staining of the SV‐40 T‐antigen in neoplastic cells, which was absent in the non‐neoplastic ones (Figure 3). Immunofluorescence staining revealed overlapping expression patterns for GATA‐3 and SV40, with both markers co‐localizing to the same neoplastic cell population. This finding suggested a role for prior BK virus infection, despite plasma BK viral DNA PCR being negative one year before tumor diagnosis. The other viral PCR assays, including EBV and CMV, were also negative.

The postoperative course was complicated by an episode of acute kidney injury attributed to calcineurin inhibitor toxicity, as well as infectious colitis due to *Clostridium difficile* , Salmonella, and norovirus.

Tumoral lesions were limited to the bladder mucosa. No adjuvant chemotherapy or radiotherapy was deemed necessary. Close monitoring by ultrasound and cystoscopy every 3 then 6 months showed no relapse and no other malignancy during the following 7 years until transition to adult transplant care. In the meantime, graft function deteriorated and required initiation of peritoneal dialysis followed by a second kidney transplant at the age of 17.5 years (6 years following cancer remission). One month following the second kidney transplantation, the patient developed a transient isolated increase in BKV viremia, in the absence of histological evidence of BKV‐associated nephropathy. Management consisted of intravenous immunoglobulin administration and modification of the immunosuppressive treatment, including conversion from everolimus to mycophenolate mofetil. The patient remained negative for BKV viremia during follow‐up with excellent graft function.

## 2 Discussion

BK virus is a polyomavirus that most people have met during childhood. Primary infection is typically asymptomatic or sub‐clinical for immunocompetent individuals, but the BK virus remains latent in the kidneys and uroepithelial cells, among other locations [4]. For individuals who are immunosuppressed, primo‐infection or secondary reactivation can cause clinically relevant infections. This situation is a well‐recognized complication of kidney transplantation, known as BK virus‐associated nephropathy.

In BK naïve transplanted patients, BK virus infection is reported to occur within the first few weeks post‐transplantation for primary infection, whereas secondary infection can be delayed for months. Pediatric recipients are particularly susceptible to develop a primo‐infection as only 50% of children show antibodies before 4 years of age, to reach 100% only at the age of 8 in the general population [4]. BK virus nephropathy is a tubulointerstitial nephritis that can mimic graft rejection and even lead to graft loss when misdiagnosed. Establishing the link between BK virus infection and deterioration of kidney function can be delicate, and graft biopsy is often needed. When nuclear inclusions are found in histological sampling, the BK virus is supposed to be involved, which is confirmed by immunohistochemistry using SV40, a polyomavirus closely related to the BK virus [5]. A reduction in immunosuppressive treatment may allow control of viral replication and a resolution of BK virus‐associated nephropathy if still reversible.

Apart from nephritis, the BK virus preferentially targets the urinary tract. Indeed, hemorrhagic cystitis is associated with BK virus infection in bone marrow transplants, and urothelial carcinoma or ureteral stenosis has been reported in kidney transplant recipients [6, 7, 8]. However, the urinary bladder is not a frequent location for carcinoma. The association of bladder urothelial carcinoma with the BK virus has been suspected in several reports of adult patients [9, 10, 11]. All of them developed bladder carcinoma after a BK virus nephropathy. Anh et al. [12] described the first pediatric case of BK virus‐associated bladder urothelial carcinoma in an 8‐year‐old kidney transplant recipient who underwent cystoprostatectomy and bilateral native nephroureterectomies, followed by cutaneous ureterostomy of the transplanted kidney. BK virus infection in immunocompromised patients has also been associated with the development of other urological malignancies, including prostatic, ureteral, and renal tumors. Emerson et al. [7] reported a case of collecting duct carcinoma arising in association with BK virus nephropathy in an adult living‐related donor renal allograft to a pediatric recipient. Similarly, Oikawa et al. [13] described a case of urothelial carcinoma originating from a ureteral graft in an adult kidney transplant recipient, diagnosed three years after BK virus‐associated nephropathy.

Solid organ transplant recipients have a substantially increased risk of malignancies compared to the general population [14], especially for virus‐related tumors, such as Epstein–Barr virus (EBV) or Human Papillomavirus (HPV). Nevertheless, the oncogenic role of the BK virus in humans remains debated, even though it has been postulated by several findings. Indeed, the incidence of urothelial carcinoma is increased in BK virus viruric recipients compared with nonviruric ones [15, 16]. Interestingly, the incidence of bladder carcinoma may also be significantly increased in immunocompetent patients, as suggested in a large study comparing immunocompetent patients with or without BK virus infection [17]. Several large‐scale studies have sought to identify a statistical association between BK polyomavirus infection and the development of urothelial tumors, which could further support a causal link; however, their conclusions remain constrained by the relatively low incidence of urothelial cancers [8, 18].

The oncogenic potential of the BK virus might be explained by the expression of the large T antigen (T‐Ag), which is a viral protein known to inactivate tumor suppressor proteins such as pRb and p53. Additional mechanisms have been proposed, including the activation of unrepaired APOBEC3 enzymes—normally regulated by p53—through polyomavirus‐encoded oncoproteins. Furthermore, infection of non‐epithelial cell types, such as fibroblasts, may contribute to a microenvironment that facilitates malignant cell migration and invasion [19].

In these different studies, a causal link cannot be proven as the BK virus is ubiquitous, and the occurrence of malignancy in immunosuppressed patients is highly independent of their BK viremic status. As Papadimitriou et al. found in their study, the most relevant evidence for the BK virus's implication in urothelial carcinoma is suggested when its DNA is found in malignant cells [20]. In our patient, tumor‐specific detection of SV40—a polyomavirus closely related to BK virus—supports an association between prior BK virus infection and the development of bladder carcinoma.

Finally, current treatment recommendations for high‐risk bladder carcinoma are relatively aggressive, and partial cystectomy is not recommended for high‐grade tumors. However, these recommendations have been devised for the adult population, which is why, because of the specific clinical situation and age of our patient, we opted for a less aggressive strategy. Moreover, a multidisciplinary oncology meeting proposed adjuvant chemotherapy, which was finally declined due to poor kidney graft function and the severe comorbidities of our patient.

Our case may be the second bladder urothelial carcinoma described in the pediatric population, and it adds further evidence of the carcinogenic potential of the BK virus in the urinary tract.

## Funding

The authors have nothing to report.

## Disclosure

The authors declare nothing to disclose.
